# Challenges in rehabilitation of patients with nontraumatic spinal cord dysfunction due to tumors: A narrative review

**DOI:** 10.1007/s00508-019-1528-z

**Published:** 2019-07-16

**Authors:** Anna Pataraia, Richard Crevenna

**Affiliations:** Department of Physical Medicine, Rehabilitation and Occupational Medicine, Medical University of Vienna, Währinger Gürtel 18–20, 1090 Vienna, Austria

**Keywords:** Spinal cord injury, Non-traumatic spinal cord dysfunction, Tumor, Rehabilitation, Barriers

## Abstract

The incidence of cancer-associated non-traumatic spinal cord dysfunction is rising due to population aging and better cancer treatment. The overall benefit of rehabilitation in specialized facilities for traumatic spinal cord dysfunction has been confirmed many times. Because of their fragility and multiple comorbidities cancer patients still face challenges to complete rehabilitation in the spinal rehabilitation facilities. In this narrative review we describe specific aspects, challenges in rehabilitation and opportunities to improve care. A literature search was performed in the PubMed database from 1 January 1978 to 30 November 2018. The focus was to find publications that discuss challenges and opportunities for rehabilitation of patients with non-traumatic spinal cord dysfunction due to a tumor. Most publications described the benefits of rehabilitation in specialized facilities. There were only few publications about survival and functional outcomes after rehabilitation for this patient population. Overall benefits including fewer complications associated with spinal cord dysfunction, less pain and depression, and better quality of life were shown. Within the past decades increasing number of publications revealed a growing interest for this group of patients. Despite major progress in cancer treatment, patients still have a limited vital prognosis and access to specialized rehabilitation units because of the concerns about the medical complexity. Patients with spinal cord tumors can benefit in areas of functionality, mood, quality of life, and survival from inpatient rehabilitation programs, in spite of the increased medical comorbidities.

## Introduction

Spinal cord injury (SCI) is a debilitating condition resulting in paralysis with associated severe consequences. Non-traumatic spinal cord dysfunction (NTSCD) is suspected to be more common than a traumatic SCI in many developed countries, with best evidence shown in publications from Canada, Australia and Norway [1–3]. A wide range of heterogeneous etiologies cause NTSCD. The most common causes are degenerative changes of the spinal column, benign and malignant tumors, vascular diseases, infections and inflammation [4–6]. The patients with NTSCD are usually older, with a typical median age of 60–65 years [1, 4, 6]. Tumors account for up to one third of all NTSCD patients admitted into spinal cord rehabilitation units (SRU) [4, 5]. With expected aging of the population in the near future the incidence of NTSCD due to tumors will also increase and potentially overwhelm healthcare services [7–9]. While most aspects of rehabilitation for traumatic spinal cord injury and NTSCD are the same, people with NTSCD due to a tumor have some unique rehabilitation issues when considering the functional deficits from the spinal cord involvement, comorbidity and life expectancy [10, 11]. The primary goal of the rehabilitation of such patients is improvement of maximum functional independence, the quality of life and a reintegration into daily life [12]. It is not surprising that rehabilitation of the patients is a great challenge. It requires a multidisciplinary approach, which includes surgical, radiation and oncological treatment [13]. Considering that, a high percentage of patients with neoplastic spinal cord compression can be discharged to home [14]. Patients with NTSCD have a better outcome when the rehabilitation is carried out in spinal cord rehabilitation units compared to a general rehabilitation, since these units have the spinal cord medical rehabilitation expertise with improved access to vital services [15, 16]. Although the early detection of tumors and advances in the oncological treatment has improved in the last decades, the full access to rehabilitative services still has barriers caused by the patient fragility and complications from concurrent medical treatment [17].

The early recognition of NTSCD with its associated neurological complications and timely submission to rehabilitation would result in better functional independence of the patients but the long-term results of rehabilitation are not depicted in newer studies. This primarily resulted from the poor survival time [17, 18]. Despite the high proportion of people with a tumor causing NTSCD, there are relatively few publications about the conditions of the ideal rehabilitation setting and there is also a lack of recommendations for rehabilitation [14, 15]. This narrative review tries to identify specific aspects and challenges in rehabilitation of patients with NTSCD due to tumors and the possibilities to improve rehabilitation.

## Methods

The narrative review includes most relevant studies regarding the rehabilitation of patients with NTSCD due to tumors (both benign and malignant), which were published in the PubMed database. The search was conducted in November 2018, the search period was restricted to 1 January 1978 to 30 November 2018 and included the following keywords: spinal cord injury, spinal cord dysfunction, tumor and rehabilitation. After assessing the most relevant studies a search of secondary sources was performed, which also included the references of initially identified articles. Only literature published in the English language and involving humans was included. The focus of this search was to identify publications that discuss challenges and possibilities for rehabilitation of patients with NTSCD due to tumors.

## General rehabilitation issues

There are numerous publications about the benefits of inpatient rehabilitation in SRU compared to general rehabilitation facilities [19, 20]. Despite that, many SRU preferentially admit SCI over those with NTSCD [20, 21]. The important aspects for this are uncertain neurological prognosis and relatively short life expectancy. Patients with limited life expectancy are managed with shorter rehabilitation hospital admissions, which focus on basic rehabilitation goals and adaptation of temporary devices [20]. Predicting neurological improvement, rehabilitation outcomes, and survival is extremely challenging and there is very little literature on this aspect [22, 23]. People with NTSCD due to tumors are generally older with more comorbidities, which can influence rehabilitation outcomes. Overall, persons with NTSCD benefit from inpatient rehabilitation [6, 20, 21, 24, 25].

## Oncological status and management

The primary goal of cancer therapy is to maintain the neurological status, reduce pain and avoid further complications [29]. Management of spinal tumors varies depending on numerous factors, such as stability of the spine, pain quality and changes in neurological status [26]. It is influenced by different treatment procedures, which include surgery, radiation therapy, and concomitant chemotherapy. The surgery should be performed in patients with spinal instability with concomitant compression of the spinal cord. After complete removal of the tumor, the survival rates increase [27, 28]. Myelopathy is a potential side effect of radiation therapy, which may appear early after treatment or with a delay of up to 2 years [31]. For some tumors, chemotherapy can also be used as an adjuvant therapy and corticosteroids are also often prescribed [32]. The side effects of corticosteroids, such as hyperglycemia, infections and impaired wound healing as well as gastrointestinal bleeding and mood disorder can have a devastating impact on the patient quality of life and the rehabilitation process [32].

## Secondary health conditions influencing rehabilitation and their management

Patients with NTSCD due to tumors experience neurological complications, such as pain, bowel and bladder dysfunction, pressure ulcers, increased skin fragility and sexual dysfunction. Some of these conditions may be further complicated due to the tumor and consequently have an influence on the rehabilitation process [33]. The most common complication of neoplastic spinal cord compression is pain [33–35] and could appear up to several months prior to neurological deficits [36]. The pain is the result of spinal cord compression, bony destruction, vertebral instability due to fast growth of the tumor or spinal nerve root compression [33]. Pain management should be adequate and pain relief can be achieved by strengthening of the body stability with different modalities, such as electrical stimulation, ultrasound and heat or cold treatment [37, 38]. Medications including non-steroidal anti-inflammatory drugs, anticonvulsants, tricyclic antidepressants, steroids, and opioids can also significantly reduce pain [39–41]. Another complication is constipation due to inadequate emptying of the bowels [12, 42]. It can be caused due to the affection of motor neurons by the tumor and may be intensified by immobility and malnutrition. An active bowel evacuation should be quickly initiated and supported by stool softener medication and laxatives combined with digital stimulation [13]. Bladder dysfunction is another common delayed complication of NTSCD and may present as symptoms, such as urgency, retention or incontinence, and frequent urinary tract infections [12, 33]. Bladder management methods include timed voiding, intermittent catheterization, and indwelling catheters [33].

The skin of patients with NTSCD can be affected by developing pressure ulcers, which can be worsened due to immobility, bladder and bowel incontinence, and malnutrition. A large proportion of this complication is preventable using appropriate techniques and education of the patient and caregivers [12]. Patients who undergo radiation treatment can develop dermatitis, hair loss, atrophy and fibrosis of the skin with loss of pigmentation [43, 44]. Patients with NTSCD can experience sexual dysfunction. This may be as a result of spinal cord injury due to cancer but sometimes it also occurs after oncological treatment [45]. The rehabilitation program should include education of patients, using assistive devices and if necessary, oral medications for sexual dysfunction [46].

## Rehabilitation issues of patients with NTSCD

Rehabilitation of patients with NTSCD due to tumors is compounded by several factors, including overall tumor burden with associated secondary depression, adverse reaction of different anti-tumor medications, sedative effect of pain relief medication, effect of radiation, and tumor-related fatigue. Some of these factors can be influenced by correction of the causes. For example, fatigue could be a result of anemia or hypothyroidism secondary to radiation. Thus, supportive care is essential and will help in optimization and improvement of the rehabilitation course [47]. Adequate pain treatment should be applied after cancer surgery. Although pain could also be secondary to neoplastic nerve/spinal cord compression and induced by radiation therapy or chemotherapy [48, 49]. Cancer patients are more susceptible to both anorexia and cachexia [50]. Depressive disorders are prevalent in cancer patients and often remain undetected and untreated [51]. Respiratory complications, wound infection or dehiscence, bleeding, as well as cerebrospinal fluid leakage are some of the side effects of oncological treatment [30]. Infections are also common and could be due to neutropenia from chemotherapy or concurrent steroid use [52]. Patients with NTSCD frequently develop deep venous thromboembolism, despite receiving anticoagulation therapy [53]. Anemia in cancer patients is multifactorial and may be caused by chronic blood loss, iron deficiency, chemotherapy-induced myelosuppression and many other factors. Typically, anemia is treated with iron substitution, erythropoietic stimulating agents or transfusion [54].

When diagnosed with cancer and spinal cord dysfunction, patients have to undergo difficult adjustments, from denial of their disability to acceptance and learning to live with a new situation [55]. Patients’ individual perception of quality of life is influenced by spiritual well-being and level of education, which should be taken into account when designing a rehabilitation program [56]. It is also important to discuss advance healthcare directives and resuscitation orders with the patients [57]. The oncological prognosis should be an essential factor for designing the goals of a rehabilitation program, even though inpatient rehabilitation has proven advantages in many aspects, such as improved survival and self-care [58]. Positive effects of rehabilitation have been shown in numerous publications [6, 20, 21, 24, 59, 60]. The majority of patients with NTSCD maintained even several months after discharge improvements in wheelchair use, ambulation, stair climbing, dressing and personal hygiene [33]. Fewer patients that completed inpatient rehabilitation programs were readmitted to the hospital as a result of medical complications [24]. Patients with less aggressive tumors who were treated with surgery and radiation and showed slowly progressing neurological symptoms and better ambulation prior to rehabilitation, had improved outcomes and survived longer with a better quality of life [59]. Patients with NTSCD due to a tumor should be assessed differently than the patients with traumatic SCI when the care support plan is being designed. Patients with cancer involvement for spinal cord dysfunction are generally older und multimorbid [61]. Rehabilitation programs for NTSCD and traumatic SCI patients are similar in many ways but some adjustments always have to be made based on concomitant cancer-related health disorders, for example fatigue or cachexia. It is also important to educate patients appropriately about bowel and bladder care, which contributes to prevention of additional skin damage and improves survival for several months [62, 63]. Skin integrity may also be affected due to concurrent malnutrition and radiation effects, which could pose significant problems, such as prolonging the rehabilitation because of pre-existing pressure sores [58, 64].

## Setting of rehabilitation and practical considerations

Authors of several studies have shown that patients with functional loss from spinal cord compression can make significant functional gains from inpatient rehabilitation from admission to discharge based on total functional independence measure scores [19–21, 60]. The majority of patients who underwent inpatient rehabilitation maintained self-care and mobility and achieved independent function 1 year after discharge [33, 62, 63]. Several studies could show that the ideal setting for rehabilitation for people with NTSCD due to tumors is a SRU [19, 24, 58]. A discussion regarding anticipated rehabilitation goals, estimated length of hospital stay, likely costs of the equipment, possible home modifications, and an estimate of ongoing care should be done. Criteria have been proposed for guiding decisions regarding the rehabilitation of people with NTSCD due to tumors and have been successfully implemented in SRUs [11, 65]. Key aspects of these rehabilitation criteria are summarized as follows:

- Main attention should be paid to the neurological status and to the degree of spinal cord damage. With increased severity of spinal cord damage, the changes of improvements in neurological function are decreased.
- The next important step in choosing the rehabilitation unit is the determination of oncological status. Prognosis varies considerably for tumors of different types.
- Secondary medical health conditions can influence patient’s ability to participate in rehabilitation and need to be optimally managed. Primarily a comprehensive evaluation and management of pain should be done.
- The patients’ social support has a major influence on planning discharge to home and should be discussed from the very beginning of the rehabilitation.

These criteria are vital to guide the decision of admitting patients with NTSCD due to tumors into SRU [6, 65, 66]. Rehabilitation-specific team and organization issues include a target length of stay for inpatient rehabilitation, based on previous studies and opinion of experts, of 4–6 weeks for those with a poor prognosis [33, 58, 64, 65]. A multidisciplinary approach with regular consultation meetings is required to determining the best treatment for a patient with NTSCD [67].

## Conclusion

In the past decades, a growing interest for NTSCD is reflected in increased publication numbers for this group of patients. Despite major progress in cancer treatment, patients with NTSCD due to a tumor still have a limited vital prognosis and access to specialized rehabilitation units. Full access to treatment in SRUs is still limited due to concerns about fragility and medical complexity associated with this diagnosis. Patients with spinal cord tumors can benefit in areas of functionality, mood, quality of life, and survival from inpatient rehabilitation programs, in spite of increased medical comorbidity from the disease process itself.
